# The National Swedish Lymphoma Register – a systematic validation of data quality

**DOI:** 10.2340/1651-226X.2024.40431

**Published:** 2024-07-10

**Authors:** Karin E. Smedby, Sandra Eloranta, Tove Wästerlid, Victor Falini, Urban Jerlström, Fredrik Ellin, Karin Papworth, Johanna Westerberg, Catharina Lewerin, Per-Ola Andersson, Hallgerdur Lind Kristjansdottir, Lena Brandefors, Charlott Mörth, Karin Hallén, Nevzeta Kuric, Amal Abu Sabaa, Björn E. Wahlin, Daniel Molin, Gunilla Enblad, Ann-Sofi Hörstedt, Mats Jerkeman, Ingrid Glimelius

**Affiliations:** a Division of Clinical Epidemiology, Department of Medicine Solna, Karolinska Institutet, Stockholm, Sweden; b Department of Hematology, Karolinska University Hospital, Stockholm, Sweden; c Regional Cancer Center South, Lund, Sweden; d Department of Oncology, Örebro University Hospital, Örebro, Sweden; e Department of Internal Medicine, Kalmar Hospital, Kalmar, Sweden; f Department of Clinical Sciences, Lund University, Lund, Sweden; g Department of Oncology, Umeå University Hospital, Umeå, Sweden; h Department of Hematology, Linköping University Hospital, Linköping, Sweden; i Section of Hematology and Coagulation, Sahlgrenska University Hospital; j Department of Internal Medicine and Clinical Nutrition, Sahlgrenska Academy, Gothenburg, Sweden; k Department of Internal Medicine, Sunderbyn Hospital, Luleå, Sweden; l Department of Oncology, Västerås Hospital, Västerås, Sweden; m Department of Oncology, Karlstad Hospital, Karlstad, Sweden; n Department of Internal Medicine, Halmstad Hospital, Halmstad, Sweden; o Department of Oncology, Gävle Hospital, Gävle, Sweden; p Department of Medicine Huddinge, Karolinska Institutet, Stockholm, Sweden; q Department of Immunology, Genetics and Pathology, Cancer Immunotherapy, and Cancer Precision Medicine, Uppsala University, Uppsala, Sweden; r Department of Oncology, Uppsala University Hospital, Uppsala, Sweden; s Department of Oncology, Skåne University Hospital, Lund, Sweden

**Keywords:** Lymphoma, validation, coverage, timeliness, exact agreement

## Abstract

**Background and purpose:**

The Swedish Lymphoma Register (SLR) was initiated in the year 2000 with the aim to monitor quality of care in diagnostics, treatment and outcome of all lymphomas diagnosed nationally among adults. Here, we present the first systematic validation of SLR records as a basis for improved register quality and patient care.

**Patients and methods:**

We evaluated timeliness and completeness of register records among patients diagnosed with lymphoma in the SLR (*n* = 16,905) compared with the National Cancer Register for the period 2013–2020. Comparability was assessed through evaluation of coding routines against national and international guidelines. Accuracy of 42 variables was evaluated through re-abstraction of data from medical records among 600 randomly selected patients diagnosed in 2016–2017 and treated across all six Swedish healthcare regions.

**Results:**

Completeness was high, >95% per year for the period 2013–2018, and >89% for 2019–2020 compared to the National Cancer Register. One in four patients was registered within 3 months, and 89.9% within 2 years of diagnosis. Registration instructions and coding procedures followed the prespecified guidelines. Missingness was generally low (<5%), but high for occasional variables, for example, those describing maintenance and consolidative treatment. Exact agreement of categorical variables was high overall (>80% for 24/34 variables), especially for treatment-related data (>80% for 17/19 variables).

**Interpretation:**

Completeness and accuracy are high in the SLR, while timeliness could be improved. Finetuning of variable registration guided by this validation can further improve reliability of register reports and advance service to lymphoma patients and health care in the future.

## Introduction

In the Nordic countries, personal identification numbers and long-standing record-keeping traditions have paved the way for high-quality register-based evaluations of health care [1]. The registers also represent powerful tools for population-based research of routine patient management, with less selection, longer follow-up and to a much lower cost than in clinical trials [2]. Malignant lymphomas represent a heterogeneous group of lymphoid neoplasms with differing morphology, molecular biology and clinical course. In the latest World Health Organization (WHO) and International Consensus Classification (ICC) [3, 4], more than 80 lymphoma subtypes are recognized, guiding clinical management and therapy. Lymphomas rank among the 10 most common malignancies worldwide and approximately 2,300 patients are diagnosed annually in Sweden. Owing primarily to improved survival, the prevalence of lymphoma survivors has risen substantially in recent years [5], and more knowledge to further understand the heterogeneity of these malignant disorders is needed.

The Swedish Lymphoma Register (SLR) was initiated in the year 2000, primarily to monitor and evaluate the quality of care in lymphoma nationally across Sweden’s six health care regions, and secondarily to form a basis for research. Cancer reporting in Sweden is mandatory by law and all cancer diagnoses are reported to the Swedish National Cancer Register (NCR) according to the International Classification of Disease (ICD) system. The SLR adds large clinical value as a basis for evaluations of routine clinical care and lymphoma-specific treatment compared to the less granular registration in the NCR. There is also an increasing need for treatment monitoring from regional and governmental authorities, patient advocates and other stakeholders in view of the rapid development of novel costly cancer therapies.

The aim of this study was to present the first reported systematic validation of variables recorded in the SLR. The validation has been carried out according to national and international recommendations [6–8] and includes the four dimensions: timeliness, completeness, comparability and validity. The aim is to improve the quality of registered variables and to facilitate continuous, reliable and up-to-date evaluation of Swedish lymphoma care, to enable competitive research and ultimately improve patient care and survival.

## Methods

### Background

The SLR records all incident primary diagnoses of lymphoma among Swedish residents aged ³18 years, with the exception of cases diagnosed at autopsy. A parallel registration is made in the NCR. At the initiation of the SLR in January 1st, year 2000, registration of data from the medical records was based on manual reporting from each hospital, and the recorded information was limited to patient and disease characteristics at diagnosis and diagnostic procedures. In 2007, the register switched to electronic registration through the INCA (Information Network for CAncer care) platform, and variables were added to capture primary treatment modalities and response. In 2010, variables were incorporated to encompass disease progression and relapse during follow-up. Additional major changes were implemented in 2019 with automated quality control checks to improve variable completeness and avoid erroneous registration of quantitative data, and allow for more flexible variable selection based on lymphoma subtype. In the same year, follow-up information was extended to include detailed subtype at relapse and relapse treatments. The SLR registration is performed by personnel employed at each diagnosing hospital, and the register records are subsequently monitored by the Regional Cancer Center (RCC) organization. Registration instructions are listed in a separate manual.

### Timeliness and completeness

We evaluated timeliness of registrations and completeness of all SLR records compared to the NCR (ICD-O/3 codes in Supplementary Table 1), for the period 2013 to 2020. Timeliness was defined as the elapsed time between the date of lymphoma diagnosis and the reporting date to the register and was assessed overall and separately for the six different health care regions in Sweden. Completeness was the extent to which all incident lymphomas reported to the NCR were also included in the SLR, taking register inclusion and exclusion criteria into account. Reporting of cancer diagnoses to the NCR is mandated by law in Sweden for all clinicians and pathologist/cytologists, and the coverage is close to 100% [9].

### Patient and variable selection for re-abstraction of medical records

Patients were sampled for re-abstraction according to a two-step procedure. Firstly, we reached out to lymphoma physicians in all seven Swedish University hospitals, and to at least two region- and county-level hospitals in each region (except Stockholm/Gotland where the University hospital manages the vast majority of all lymphoma patients), to nominate a physician or nurse to perform the re-abstraction locally (22 hospitals were selected, representing all six regions, Supplementary Table 1). The target sample size was set to represent 15% (*n* = 600) of the full patient population diagnosed in 2016 and 2017. Patients treated at the selected hospitals were randomly sampled according to a Probability Proportional to Size (PPS) principle based on the proportion of patients diagnosed in each hospital during the year before (2015) (Supplementary Table 2). We included patients registered with all lymphoma subtypes except primary cutaneous lymphomas, since these types have been evaluated separately [10]. The re-abstraction was performed in 2021–2022.

Forty-two out of 57 available register variables were selected for re-abstraction representing important and relevant information regarding patient and disease characteristics, diagnostic and staging procedures and primary treatment (more administrative variables and some blood test results were excluded). Staging was performed according to the Lugano and Musshoff classifications [11, 12]. For the purpose of the re-abstraction, a copy of the electronic registration form containing the 42 selected variables was created on the INCA platform. To improve evaluation of the qualitative content of a few variables, pre-specified information was abstracted separately for these. The separate abstraction included if performance status at diagnosis was specified in the medical records (yes/no), the exact number of intrathecal injections administered as central nervous system (CNS) prophylaxis, and if additional primary treatment modalities and/or clinical trial protocols were used that were not specified in the electronic form. The re-abstraction was blinded; that is, data was abstracted from the medical records without access to the data that was originally recorded in the SLR.

### Validity

To evaluate the accuracy and validity of the data recoded in the SLR, the re-abstracted data was compared with the originally recorded data to calculate the exact data agreement. Exact agreement corresponds to the proportion of patients for whom the data recorded in the SLR is the same as in the re-abstracted data. We chose to include missing observations in the calculation of exact agreement to account for the realistic event that information could in some instances be missing in the medical records or in the original register records. Strength of agreement was measured with Cohen’s kappa score (К, with 95% confidence intervals, CI) for categorical variables. A К of 0.61–0.80 was interpreted as substantial agreement, and a score of 0.81–1.00 as almost perfect [13]. For numerical variables (including dates), the Pearson correlation coefficient was used. Throughout the review, we have accounted for coding instructions and logical relationships between variables (e.g. validity of treatment data was restricted to patients selected for the various treatment modalities) and relevance of variables depending on lymphoma subtype.

### Comparability

To ensure that registrations and coding of new lymphoma cases into the SLR are robust and comparable over time within as well as outside of Sweden, we reviewed diagnosis codes, registration practices, and supporting documents used by monitors at each RCC (six in total, representing each Swedish health care region). The register monitors check new registrations before they are entered into the system, and communicate with the hospitals if necessary, to resolve unclarities. Reviewed data sources included national and international coding guidelines, electronic report forms, registration manual and clinical management guidelines (available at www.cancercentrum.se) according to their status in November 2022. The main focus of this work was to ensure that diagnosis codes and dates are defined and recorded in a consistent way in each region and associated hospitals, as recommended by Bray and Parkin [7]. The work was coordinated by ASH and VF who were in contact with the register monitors.

The study was approved by the regional ethics review board in Stockholm (Dnr 2021-01079).

## Results

### Timeliness

**Figure 1:**
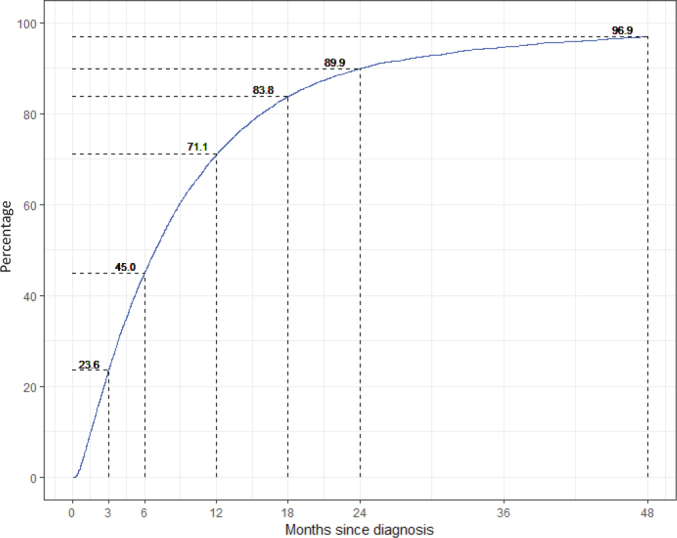
Completeness of the Swedish Lymphoma Register (SLR) compared with the Swedish National Cancer Register by time since diagnosis (in months) for the period 2013–2020.

During the period 2013–2020, the median time from diagnosis to registration was 6.8 months among all patients (*n* = 16,905) in the SLR. Approximately one in four (23.6%) were registered within 3 months, 89.9% were registered within 2 years, and 96.9% within 4 years (Figure 1). The proportions were largely similar across health care regions (Table 1).

**Table 1:** Timeliness of reporting to the Swedish Lymphoma Register (SLR) by health care region of 16,905 patients diagnosed during the period 2013–2020.

Health care region	Registered after 3 months *n* (%)	Registered after 12 months *n* (%)	Registered after 24 months *n* (%)	Registered after 48 months *n* (%)
Stockholm/Gotland	517 (14.7)	2201 (62.4)	2985 (84.6)	3305 (93.7)
Middle Sweden	1171 (32.0)	2946 (80.5)	3381 (92.4)	3574 (97.7)
Southeast	984 (55.1)	1634 (91.5)	1734 (97.1)	1772 (99.2)
South	421 (12.5)	1963 (58.4)	2790 (83.0)	3211 (95.5)
West	721 (22.6)	2393 (75.0)	3043 (95.4)	3164 (99.2)
North	224 (13.8)	1034 (63.9)	1474 (91.1)	1591 (98.3)
**Total**	**4038 (23.6)**	**12171 (71.1)**	**15407 (89.9)**	**16617 (96.9)**

Total numbers are highlighted in bold.

### Completeness

Nationally, completeness of identification of lymphoma patients in the SLR compared to the NCR was >95% each year for the period 2013–2018, but lower for 2019 (91.5%) and 2020 (89.3%) (Table 2). All six health care regions had a completeness of 90% and above for the period 2013–2018.

**Table 2:** Completeness of the Swedish Lymphoma register (SLR) compared to the National Cancer Register (%), by health care region and year for the period 2013–2020.

Year of diagnosis	Stockholm/Gotland	Middle Sweden	Southeast	South	West	North	Total
2013	97.0	100	100	99.8	99.7	100	**99.3**
2014	97.9	100	100	99.5	98.8	100	**99.2**
2015	96.4	99.3	96.3	99.0	99.2	100	**98.4**
2016	97.6	99.8	95.9	97.4	98.3	99.5	**98.1**
2017	93.3	98.5	94.9	98.8	97.3	99.5	**96.9**
2018	92.0	95.2	94.2	96.0	98.5	100	**95.5**
2019	88.3	93.3	94.4	83.3	96.6	99.5	**91.5**
2020	91.9	93.3	85.2	79.1	90.5	99.4	**89.3**

Total numbers are highlighted in bold.

### Validity

**Figure 2:**
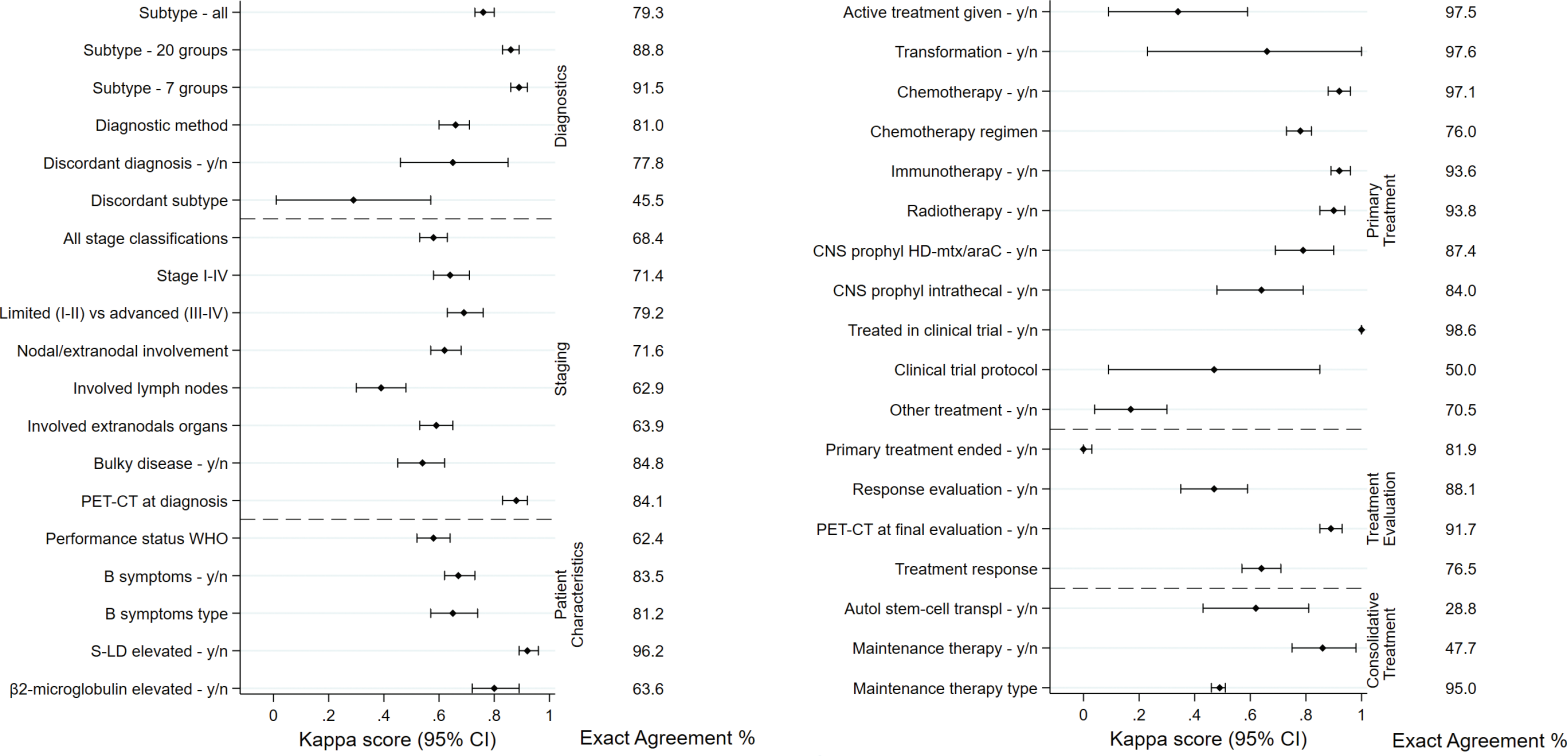
Kappa scores of the correlation between registered and re-abstracted data for variables related to diagnostics, staging and patient characteristics (A), and primary treatment, treatment evaluation and consolidative treatment (B). Exact agreement estimates including missing are shown to the right.

Among all 600 patients evaluated against medical records, 599 were confirmed to have a lymphoma diagnosis whereas one had been diagnosed with acute lymphoblastic leukemia. This patient was subsequently excluded from further analysis. In the cohort of confirmed lymphoma patients, median age was 69 years (range 18 to 95 years) and male sex was slightly overrepresented (*n* = 328, 54.8%). Missingness was low (<5%) in the SLR for most of the variables (31/42, 74%) assessed (Table 3). One variable (revised treatment decision) was ambiguous to complete retrospectively and further results are therefore not shown for this variable. Recorded lymphoma subtype diagnoses spanned across 50 different entities. The exact agreement of lymphoma subtype when all subtypes were considered, was 79.3% (К = 0.76, 95% CI: 0.73–0.80, Figure 2). When subtypes were collapsed into 20 broader entities (19 subtypes plus missing as one category) (Table 4, Supplementary Table 3), agreement rose to 88.8% (К = 0.86, 95% CI: 0.83–0.89), and when collapsed to seven groups, it rose to 91.5% (К = 0.89, 95% CI: 0.86–0.92). At the 20 subtypes-level, the proportion of patients confirmed to have the same subtype in the medical records as in the register was lowest among poorly defined subtypes such as low-grade B-cell lymphoma not otherwise specified (NOS), T/NK-cell lymphomas NOS and other/unspecified lymphomas (Table 4). Disagreement was mostly due to classification differences of related subtypes for example, a registered diagnosis of high-grade B-cell lymphoma was categorized as diffuse large B-cell lymphoma in the medical records review.

**Table 3:** Overview of variables in the Swedish Lymphoma Register (SLR) selected for re-abstraction through medical records in the assessment of validity, and number and proportion of missing values. The denominator for each variable reflects the number of patients for whom this variable should be recorded.

SLR variable	Missing values in SLR *n* (%)	Missing values in dataset re-abstracted from medical records *n* (%)
**Diagnostics**		
Diagnosis date	0/599 (0)	1/599 (0.2)
Subtype – all	0/599 (0)	1/599 (0.2)
Diagnostic method	0/599 (0)	0/599 (0)
Discordant diagnosis – y/n	101/599 (16.9)	23/599 (3.8)
Discordant subtype	0/17 (0)	0/19 (0)
**Staging**		
Stage according to Ann Arbor*	2/510 (0.4)	3/528 (0.6)
Stage according to Musshoff*	0/79 (0)	0/61 (0)
Nodal/extranodal involvement	22/599 (3.7)	11/599 (1.8)
Involved lymph nodes	48/166 (28.9)	0/159 (0)
Involved extranodal organs	29/333 (8.7)	0/360 (0)
Bulky disease – y/n	0/599 (0)	4/599 (0.7)
PET-CT at diagnosis – y/n	10/508 (2.0)	50/509 (9.8)
**Patient characteristics**		
Performance status (WHO)	0/592 (0)	3/567 (0.5)
B-symptoms – y/n	0/599 (0)	3/599 (0.5)
B-symptoms type	5/196 (2.6)	0/165 (0)
S-LD elevated – y/n	6/599 (1.0)	15/599 (2.5)
S-LD value	1/586 (0.2)	1/587 (0.2)
Beta2microglob elevated – y/n	56/237 (23.6)	16/225 (7.1)
**Primary treatment**		
Active treatment given – y/n	0/599 (0)	0/599 (0)
Revised treatment decision – y/n	204/599 (34.1)	77/599 (12.9)
Treatment start date	0/580 (0)	0/580 (0)
Transformation – y/n	1/358 (0.3)	6/344 (1.7)
Subtype of transformation	0/3 (0)	0/5 (0)
Chemotherapy – y/n	0/594 (0)	2/581 (0.3)
Chemotherapy regimen	0/471 (0)	31/463 (6.7)
Immunotherapy – y/n	1/594 (0.2)	18/581 (3.1)
Immunotherapy type	1/415 (0.2)	50/398 (12.6)
Radiotherapy – y/n	2/594 (0.3)	15/581 (2.6)
Radiotherapy, dose	0/117 (0)	0/117 (0)
CNS prophylaxis, HD-MTX/ARAC – y/n	23/286 (8.0)	0/284 (0)
CNS prophylaxis, intrathecal – y/n	28/286 (9.8)	1/284 (0.4)
Treated in a clinical trial – y/n	2/594 (0.3)	6/581 (1.0)
Clinical trial protocol	0/6 (0)	1/6 (16.7)
Other treatment – y/n	116/593 (19.6)	0/581 (0)
**Treatment evaluation**		
Primary treatment ended – y/n	79/594 (13.3)	0/581 (0)
Treatment end date	0/475 (0)	0/475 (0)
Response evaluation – y/n	4/593 (0.7)	9/581 (1.5)
PET-CT at final evaluation – y/n	17/594 (2.9)	4/581 (0.7)
Treatment response	1/463 (0.2)	3/480 (0.6)
**Consolidative treatment**		
Autologous stem cell transplant – y/n**	347/503 (69.0)	0/501 (0)
Maintenance therapy – y/n	137/271 (50.5)	0/248 (0)
Maintenance therapy type	0/24 (0)	0/21 (0)

* The two classification systems Musshoff (for primary extranodal lymphomas) and Ann Arbor (all other lymphomas) were collapsed in further analyses but are shown separately here.

** a third response alternative was ‘planned but not performed’ was recategorized as ‘no’. HD-MTX/ARAC = high-dose methotrexate/cytarabine (intravenous), CNS = Central Nervous system, S-LD = serum-lactate dehydrogenase, y = yes, n = no.

**Table 4:** Distribution of registered lymphoma subtypes in the patient sample selected for assessment of accuracy in the Swedish Lymphoma Register (SLR), and number of cases confirmed to have the same subtype in the medical record review. Here, the subtypes are collapsed into 19 groups.

Lymphoma subtype	No (%) of patients in SLR (total *n* = 599)	No (%) of cases confirmed in re-abstraction
Diffuse large B-cell lymphoma (DLBCL)	227 (37.9)	222/227 (97.8)
Follicular lymphoma (FL)	84 (14.0)	81/84 (96.4)
Classical Hodgkin Lymphoma (cHL)*	68 (11.4)	62/68 (91.2)*
Mantle cell lymphoma (MCL)	48 (8.0)	47/48 (97.9)
Mucosa-associated lymphoid tissue (MALT)	30 (5.0)	25/30 (83.3)
lymphoma		
Lymphoplasmacytic lymphoma (LPL)	26 (4.3)	24/26 (92.3)
T/NK-cell lymphoma, NOS**	12 (2.0)	8/12 (66.7)**
Low-grade B-cell, NOS	11 (1.8)	5/11 (45.5)
Angioimmunoblastic T-cell lymphoma (AITL)	10 (1.7)	9/10 (90.0)
Nodal Marginal zone lymphoma (MZL)	9 (1.5)	6/9 (66.7)^Ϯ^
Peripheral T-cell lymphoma (PTCL), NOS	8 (1.3)	5/8 (62.5)
Hairy cell leukemia (HCL)	8 (1.3)	8/8 (100)
Anaplastic large cell lymphoma (ALCL)	7 (1.2)	4/7 (57.1)
Nodular lymphocyte-predominant Hodgkin lymphoma (NLPHL)	6 (1.0)	6/6 (100)
Small lymphocytic lymphoma (SLL)	4 (0.7)	2/4 (50.0)^ϮϮ^
Burkitt lymphoma (BL)	3 (0.5)	3/3 (100)
Primary mediastinal B-cell lymphoma (PMBL)	3 (0.5)	2/3 (66.7)
Splenic Marginal zone lymphoma (MZL)	3 (0.5)	2/3 (66.7)
Other/unspecified lymphomas^§^	32 (5.3)	8/32 (0.25)^§^

NOS = not otherwise specified.

* Four patients registered with cHL were categorized as NLPHL at medical records review.

** Four patients registered with T-NK-cell lymphoma NOS were categorized as PTCL or other, unspecified at medical records review.

Ϯ Three patients registered with nodal MZL were categorized as other MZL or LPL at medical records review.

ϮϮ Two patients registered with SLL were categorized as low-grade B-cell lymphoma unspecified at medical records review.

§ Patients registered with unspecified subtypes were in most instances categorized as more specific related subtypes (DLBCL, FL, BL, SLL, SMZL) at medical records review.

Exact agreement and kappa scores were generally somewhat lower for variables related to disease characteristics at diagnosis compared with variables related to treatment. Among disease characteristics, disease stage, nodal/extranodal involvement and specific nodal and extranodal locations had exact agreements of 68.4, 71.6, 62.9, and 63.9%, respectively, whereas diagnostic methods (e.g. type of biopsy) and having performed an FDG-PET-CT scan had higher values (81.0 and 84.1%, respectively) (Figure 2). Considering stage at the level of I–IV, most discrepancies were found among patients registered with early-stage (I–II) reclassified as advanced-stage disease upon medical records review (Supplementary Table 4). Performance status had an exact agreement of 62.4%, and a specific performance status value according to WHO had only been noted in the medical records for a minority of the patients (*n* = 253, 42.2%).

Regarding treatment, for the variables chemotherapy yes/no and regimen, intravenous (iv) CNS prophylaxis yes/no, immunotherapy yes/no and radiotherapy yes/no, both exact agreements and kappa scores were high (exact agreement >76%, К > 78). Other treatment variables such as autologous stem-cell transplant (ASCT) and maintenance therapy had lower exact agreement and kappa scores (Figure 2). Here, the lower values were likely due to considerable missingness for these variables (missing for ASCT 68.9%, for maintenance therapy 60.6%, Table 3). When missing data was excluded, exact agreement rose to 92.2% for ASCT, and 95.8% for maintenance therapy (data not shown). Among patients registered to have received intra-thecal CNS prophylaxis (*n* = 25), about half (*n* = 13) received at least four injections (which was the minimum required number to record receipt of CNS prophylaxis according to the register instructions), 25% (*n* = 6) received three injections, whereas the remaining patients received fewer (1 or 2) injections. Overall, exact agreement values were broadly consistent across regions (selected variables shown in Supplementary Table 5).

**Figure 3:**
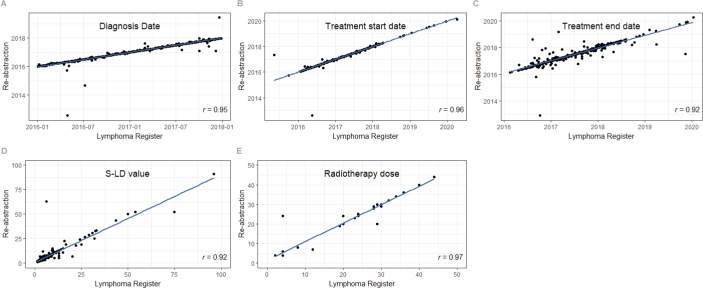
Pearson correlation between registered and re-abstracted data for dates of diagnosis, start and end of treatment as well as serum-lactate dehydrogenase (S-LD) value and radiotherapy dose in the Swedish Lymphoma Register (SLR). The *r* value represents the correlation coefficient.

For numerical variables including diagnosis date and treatment start and end dates, correlations were high (*r* = 0.95, 0.96, and 0.92, respectively) (Figure 3). This was also noted for serum-lactate dehydrogenase (S-LD) level at diagnosis (*r* = 0.92) and radiotherapy dose (*r* = 0.97).

### Comparability

Inclusion- and exclusion criteria for registration in the SLR, variable definitions and coding instructions were reviewed across supporting documents, electronic registration forms and national care programs for lymphoma management (seven documents, one for each group of related lymphoma subtypes, www.cancercentrum.se) and were found to be consistent. We could establish that Register monitors at each RCC use coding and classification instructions recommended by the International Classification of Diseases for Oncology (ICD-O) and WHO to identify new primary diagnoses of lymphoma [14]. We further confirmed consistent use of diagnosis date as the date of the first tissue sampling that resulted in a lymphoma diagnosis (or strong suspicion) as recommended for all incident primary cancers nationally [15], and awareness of the distinction between a primary lymphoma diagnosis and progression, relapse, or transformation, as defined by the European Network of Cancer Registries [16]. We found that adherence to these guidelines is secured through regular meetings among regional monitors, and that the monitors also re-evaluate pathology reports together with registration personnel at the hospitals as necessary. We moreover noted that an additional separate coding system is used in the SLR for detailed lymphoma subtype to account for the classification of lymphoproliferative disorders according to WHO and ICC [3, 4].

## Discussion

The SLR is an important national resource for comprehensive evaluation of quality of care and population-based research of lymphomas. For the first time, we performed a systematic validation of the SLR including the four dimensions of completeness, timeliness, comparability and validity. Completeness was high (>95%) for most of the studied period, but relatively slow registration of new cases and low timeliness resulted in a lower completeness for the last few years (90%). Validity was high for the majority of the variables assessed, with a low number of missing values and high exact agreement. In general, treatment variables had higher exact agreement than variables describing diagnostic characteristics, but key variables describing diagnostic procedures such as use of PET-CT also had high accuracy. We conclude that these data can be safely used as a basis for care evaluations and strategic decisions, comparisons over time and for the purpose of competitive research in lymphoma, with the goal to improve patient outcomes.

There are only a few similar nationwide lymphoma registers established globally apart from those existing in the Nordic countries and in the Netherlands [17–19]. The Danish lymphoma register (LYFO) was validated for completeness against the Danish cancer register for the period 2000–2011, and the coverage was found to be high, 94.9%, similar to the >95% seen in the SLR [17]. Eleven key variables were further evaluated in a patient subset. Both completeness and accuracy (measured as positive predictive values) were high, but lower for, for example, disease stage (in particular limited stage), in comparison to treatment, similar to the present results for the SLR. Other high-quality cohorts of lymphoma management in clinical routine also exist, such as the French REALYSA cohort [20], the hematological malignancy research network in United Kingdom [21] and the Lymphoma Epidemiology of Outcomes (LEO) cohort in the US [22]. However, the population-based coverage, and the possibilities to link to other nationwide register-based data sources are unique aspects of registers held in the Nordic countries.

Other Swedish quality-of-care registers of cancer, specifically of breast [23], prostate [24], esophageal/gastric [25], kidney [26] and colorectal [27] cancers, and other national cancer registers [28, 29], have been validated according to the same principles as here [7, 8]. Similar to the SLR, the other validated Swedish cancer quality of care registers [23–25] were found to have a very high completeness (>95%) and generally high accuracy of recorded variables, but timeliness was superior to that of the SLR for breast, prostate, kidney and colorectal cancers, with >90% of the cases recorded within 12 months instead of 24 months in the SLR. A lower timeliness of registrations for lymphomas than for common solid cancer forms could perhaps be explained by a larger clinical heterogeneity of lymphomas and varying need for active treatment, and thus a more decentralized management. Still, to maintain the relevance of the SLR for regional and national follow-up, timeliness should be improved at all hospitals where lymphomas are diagnosed and treated. The ambition of Swedish health register authorities to reduce the need for manual registration by implementing mechanisms for automated electronic data transfer [30, 31] will likely improve timeliness as well as accuracy. However, this requires the construction and use of structured medical records and ideally also coordinated medical record systems, which will take time to establish.

In the evaluation of comparability of coding of new lymphoma cases into the SLR, diagnosis dates and coding of progression, relapse, and transformation, we conclude that supporting documents are consistent and that national and international guidelines are followed and well communicated across regions in the RCC organization. Hence, the prerequisites for comparability of statistics of lymphoma incidence over time and across regions are fulfilled.

### Validity and potential sources of error

Missingness was low for most variables, including key diagnostic and prognostic factors like lymphoma subtype, stage, S-LD, dates of diagnosis, start and end of treatment, and for chemotherapy and immunotherapy regimens. Similarly, the accuracy of variables for important dates (diagnosis date, treatment start and end dates) and treatment administration was also generally high. Exact agreement for lymphoma subtype could have been expected to be even higher than the actual estimate (79.3%), but the estimate increased when subtypes were collapsed into broader categories of related subtypes. We speculate that the retrospective assessment of lymphoma subtype could inadvertently have been influenced by availability of more recent information in the medical records at the time of re-abstraction. The fact that agreement was lower for initially unspecified lymphoma types could support this theory. In addition, low accuracy was noted for consolidative treatment modalities like ASCT, which was however largely explained by missing values. Since the electronic register platform did not require all records to be completed for each patient during the period 2016–2017, we consider it likely that missingness for these rare treatments reflects that the treatments were not given.

### Future developments/consequences of the validation study

For several register variables, the present study provides concrete guidance for immediate register improvements. These include improvements of technical solutions (e.g. mandatory variable completion, automated quality checks), and supporting information in the electronic form and separate manual to facilitate standardized recording. In addition, the register organization needs to continue to find flexible solutions for variable modification and systematic data validation as lymphoma management and therapies will continue to evolve. In this process, close communication with working groups responsible for national care programs, and with lymphoma physicians and nurses managing lymphoma patients in Sweden is crucial. Furthermore, hospital and clinic heads where lymphoma patients are diagnosed and treated should work to facilitate use of structured medical records where key register variables are specified. Another important addition in the future will be registration of patient-reported outcomes, ideally through an existing national digital health care communication system.

For evaluating the quality of care and for research purposes, data in the SLR can be linked to other nationwide population and health registers maintained by Statistics Sweden and the National Board of Health and Welfare, to complement information on, for example, hospital admissions, drug prescriptions and socioeconomic factors [32–34]. Furthermore, comparisons to expected rates of health and disease in the general population can be performed through identification of age- and sex-matched comparator subjects in the population register [35]*.* Currently, a data linkage based on the SLR and more than eight other nationwide health care registers is in place forming the LymphomaBase linkage. Examples of studies from this large database are investigations of late effects of lymphoma treatments, including secondary malignancies [36, 37], fertility and childbearing [38, 39] and studies investigating patterns of relapse and survival [40, 41]. Data in the SLR can also be linked to databases of biological biospecimens such as those in local and national biobanks [42, 43]. For specific clinical research questions, data can be added through medical records review (e.g. details on radiation targets [44] or relapse locations [45]). Collaboration and pooling of data from other Nordic countries [40] and other international databases [46, 47] provide possibilities of investigations of rare lymphoma subtypes.

### Strengths and limitations

Strengths of this validation of the SLR include the long period of evaluation of completeness and timeliness (2013–2020), the relatively large data set for blinded evaluation of accuracy against medical records, the large number of variables assessed and the multi-modal validation approach. Limitations include the exclusion of primary cutaneous lymphomas in the assessment of accuracy, although completeness of these rare subtypes has been evaluated previously [10]. For pragmatic reasons, we mainly used internal validators employed at the respective hospitals to carry out the re-abstraction of data from medical records, although they were blinded to the originally recorded data. There’s a small risk that they were responsible for patient management and original SLR recording in a few instances. However, the risk of bias was deemed to be small since registrations at most hospitals are not routinely performed by physicians, and since lymphomas are common cancer forms, typically managed and recorded by several doctors at each hospital. The robustness of the results across health care regions further limits this concern. Another limitation is the fact that the validation was carried out in a recent period (2016–2017). It is thus uncertain if the results are representative of earlier periods of registration. Future validations should ideally include both early and late periods.

## Conclusions

This first systematic validation of the SLR shows high completeness, low missingness and high data accuracy for most of the evaluated variables. The SLR is a robust and comprehensive national resource to address the role of clinical, tumor, treatment and outcome factors in lymphoma prognosis and survivorship. The register thus provides a powerful tool for evaluations of quality of care and real-world research of national and international interest. The findings from this study will guide further improvements and increase register relevance for lymphoma patients and health care in the future.

## Supplementary Material

The National Swedish Lymphoma Register – a systematic validation of data quality

## Data Availability

The data used for this study is protected by GDPR to preserve individual patient integrity. Access can only be provided within the context of the obtained ethical approval and approval from the CPUA.
