# Supplementary material for: The National Swedish Lymphoma Register – a systematic validation of data quality
Source: Acta Oncol. 2024 Jul 10;63:40431. doi: 10.2340/1651-226X.2024.40431 (PMC11332524; doi:10.2340/1651-226X.2024.40431)
Supplement: The National Swedish Lymphoma Register – a systematic validation of data quality [file AO-63-40431-s1.pdf]

Supplementary material has been published as submitted. It has not been copyedited, or typeset by Acta Oncologica

Supplementary Table 1. International Classification of Diseases for Oncology, 3rd edition (ICD-O/3) codes used for matching of the Swedish Lymphoma Register to the National Cancer Register in the evaluation of completeness.

Broad lymphoma subtype	ICD-O/3 codes
Precursor B and T/NK-cell neoplasms	97273, 97283, 97293, 98273
Mature B-cell lymphomas	95913, 95973, 96703,96713, 96733, 96783, 96793, 96803, 96873, 96883, 96893, 96903, 96913, 96953, 96983, 96993, 97123, 97353, 97373, 97383, 97613, 97661, 99403
Mature T/NK-cell lymphomas	97003, 97013, 97023, 97053, 97083, 97093, 97143, 97163, 97173, 97183, 97193, 97263, 98313, 99483
Hodgkin lymphomas	96503, 96513, 96523, 96533, 96593
Immune-deficiency associated lymphoproliferative malignancies	99701, 99711, 99713
Unspecified lymphomas	95903, 95913, 959131, 959133, 959135, 959136

Supplementary Table 2. Hospitals participating in the assessment of validity, and number and proportion of patients registered in the Swedish Lymphoma register in 2015 guiding the selection of patients for validation in each region.

Health care region	Participating hospitals	No and proportion of patients registered in 2015 n (col %)	No and proportion of patients selected for validation n (col %)
**Stockholm/Gotland**	2 (Karolinska Solna + Huddinge)	276 (19.0%)	114 (19.0%)
**Middle Sweden**	6 (Akademiska Uppsala, Örebro, Falun, Gävle, Västerås, Karlstad)	348 (24.0%)	144 (24.0%)
**Southeast**	4 (Linköping, Kalmar, Jönköping, Västervik)	152 (10.5%)	63 (10.5%)
**South**	3 (Skånes Universitetssjukhus, Halmstad, Helsingborg)	271 (18.7%)	112 (18.7%)
**West**	4 (Sahlgrenska Göteborg, Borås, Uddevalla, Skövde)	279 (19.2%)	115 (19.2%)
**North**	3 (Umeå, Sunderbyn, Skellefteå)	126 (8.7%)	52 (8.7%)

Supplementary Table 3. Overview of lymphoma subtypes registered in the Swedish Lymphoma Register at the time of assessment of validity, in broad groups of 7 and 20 subtype categories, and all detailed subtypes according to the World Health Organization (WHO) classification, 4th edition. Primary cutaneous lymphomas are not listed since these were excluded from the validity assessment.

7 subtype categories	20 subtype categories*	All registered subtypes according to WHO 4th edition
Diffuse large B-cell lymphoma	Diffuse large B-cell lymphoma	Diffuse large B-cell lymphoma, not otherwise specified
		Diffuse large B-cell lymphoma associated with chronic inflammation
		EBV-positive diffuse large B-cell lymphoma in the elderly
		Large-cell lymphoma intermediate between diffuse large B-cell and Burkitt lymphoma
		Large-cell lymphoma intermediate between diffuse large B-cell and Hodgkin lymphoma
		ALK-positive diffuse large B-cell lymphoma
		Large B-cell lymphoma in multicentric Castleman's disease
		Plasmablastic lymphoma
		Intravascular large B-cell lymphoma
		Primary effusion lymphoma
		T-cell/histiocytic large B-cell lymphoma<br>Follicular lymphoma grade 3B
		Primary CNS lymphoma
	Primary mediastinal large B-cell lymphoma	Primary mediastinal large B-cell lymphoma
Follicular lymphoma	Follicular lymphoma	Follicular lymphoma grade 1
		Follicular lymphoma grade 2
		Follicular lymphoma grade 3
		Follicular lymphoma grade 3A
		Follicular lymphoma not otherwise specified
Mantle cell lymphoma	Mantle cell lymphoma	Mantle cell lymphoma<br>Mantle cell lymphoma, in situ
Indolent B-cell lymphomas excluding follicular lymphoma	Mucosa-associated lymphoid tissue (MALT) lymphoma	Mucosa-associated lymphoid tissue (MALT) lymphoma
	Nodal marginal zone lymphoma	Nodal marginal zone lymphoma
	Splenic marginal zone lymphoma	Splenic marginal zone lymphoma
	Small lymphocytic lymphoma	Small lymphocytic lymphoma
	Lymphoplasmacytic lymphoma	Lymphoplasmacytic lymphoma
		Waldenström's macroglobulinemia
	Hairy-cell leukemia	Hairy-cell leukemia
		Hairy-cell leukemia-variant
	Low-grade B-cell lymphoma, unspecified	Diffuse low-grade B-cell lymphoma of the spleen
		Low-grade B-cell lymphoma, not otherwise specified
Classical Hodgkin lymphoma	Classical Hodgkin lymphoma	Hodgkin lymphoma, nodular sclerosis
		Hodgkin lymphoma, mixed cellularity
		Hodgkin lymphoma, lymphocyte rich
		Hodgkin lymphoma, lymphocyte depleted
		Classical Hodgkin lymphoma unspecified
		Hodgkin lymphoma, not otherwise specified
Aggressive T-cell lymphoma	Angioimmunoblastisc T-cell lymphoma	Angioimmunoblastisc T-cell lymphoma
	Anaplastic large-cell lymphoma	Anaplastic large-cell lymphoma, ALK positive
		Anaplastic large-cell lymphoma, ALK negative
		Implant-associated anaplastic large-cell lymphoma
	Peripheral T-cell lymphoma, unspecified	Peripheral T-cell lymphoma, not otherwise specified
	T/NK-cell lymphoma, other	Aggressive NK-cell leukemia
		Adult T-cell leukemia/lymphoma
		Blastistic NK-cell lymphoma
		Enteropathty-associated T-cell lymphoma
		Monomorphic epitheliotropic intestinal T-cell lymphoma
		Hepatosplenic T-cell lymphoma
		NK/T-cellslymphoma, nasal type
		Subcutaneous panniculitis-like T-cell lymphoma
		Blastic aggressive T-cell lymphoma unspecified
Other		Granular lymphatic leukemia
		Chronic NK-lymphoproliferative disease
		T-prolymphocyte leukemia
		Small cell indolent T-cell lymphoma
	Burkitt lymphoma	Burkitt lymphoma
	Nodular lymphocyte-predominant Hodgkin lymphoma	Nodular lymphocyte predominant Hodgkin lymphoma
	Other/unspecified lymphomas	B-cell lymphoma, not otherwise specified

	High-grade B-cell lymphoma, not otherwise specified
	High-grade lymphoma, not otherwise specified
	Low-grade lymphoma, not otherwise specified
	Non-Hodgkin lymphoma, not otherwise specified
	Malignant lymphoma, not otherwise specified
	Post-transplant lymphoproliferative disease
	Lymphomatoid granulomatosis

* in addition to those listed, an extra category for missing information on subtype was added in the analyses

Supplementary Table 4. Number of patients with lymphoma stage I through IV at diagnosis according to the Swedish Lymphoma Register (SLR) records (rows) and according to the medical records review (columns).

**Stage according to SLR**	**Stage according to medical records review**				
	**I**	**II**	**III**	**IV**	Missing
**I**	74	10	4	23*	4
**II**	7	73	3	24	1
**III**	2	4	72	18	9
**IV**	4	14	12	197	11
Missing	6	2	1	21	3

* Nine of 23 cases (40%) represented Primary CNS lymphomas

Supplementary Table 5. Exact agreement (including missing) by health care region for selected variables.

Variable	Stockholm /Gotland	Middle Sweden	Southeast	South	West	North
**Subtype - all**	0.84	0.72	0.86	0.86	0.75	0.79
**Subtype – 20 groups**	0.91	0.86	0.94	0.91	0.87	0.87
**Diagnosis date**	0.66	0.67	0.86	0.69	0.68	0.67
**Diagnostic method**	0.88	0.81	0.81	0.80	0.72	0.88
**Stage I-IV**	0.77	0.8	0.71	0.72	0.79	0.83
**Performance status WHO**	0.58	0.75	0.63	0.79	0.54	0.69
**PET-CT at diagnosis**	0.96	0.98	0.96	0.88	0.93	0.98
**S-LD elevated – y/n**	0.95	0.96	0.97	0.94	0.98	1
**Active treatment given – y/n**	1	0.97	0.98	0.94	1	0.96
**Treatment start date**	0.85	0.78	0.85	0.81	0.86	0.82
**Chemotherapy – y/n**	0.96	0.97	1	0.97	0.97	0.98
**Chemotherapy regimen**	0.92	0.83	0.79	0.79	0.78	0.62
**Immunotherapy – y/n**	0.98	0.97	0.98	0.94	0.95	1
**Radiotherapy – y/n**	0.96	0.96	0.94	0.97	0.96	1
**Treatment end date**	0.69	0.57	0.73	0.63	0.65	0.62
**PET-CT at final evaluation**	0.96	0.96	0.91	0.94	0.96	0.94
**Treatment response**	0.81	0.86	0.73	0.78	0.73	0.83
**Maintenance therapy – y/n**	0.90	0.97	1	0.95	1	1

S-LD= serum-lactate dehydrogenase, y=yes, n=no
